# Urox containing concentrated extracts of *Crataeva nurvala* stem bark, *Equisetum arvense* stem and *Lindera aggregata* root, in the treatment of symptoms of overactive bladder and urinary incontinence: a phase 2, randomised, double-blind placebo controlled trial

**DOI:** 10.1186/s12906-018-2101-4

**Published:** 2018-01-31

**Authors:** Niikee Schoendorfer, Nita Sharp, Tracey Seipel, Alexander G. Schauss, Kiran D. K. Ahuja

**Affiliations:** 1 School of Medicine, University of Queensland, Herston, Australia; 2 Occidental Medicine Department, Endeavour College of Natural Health, Brisbane, Queensland Australia; 3 Seipel Group, Brisbane, Australia; 4 Bio5 Institute, Office of Research, Discovery and Innovation, and College of Science, University of Arizona; and, AIBMR Life Sciences, Seattle, Washington USA; 5 School of Health Sciences, University of Tasmania, Locked Bag 1322, Launceston, Tasmania 7250 Australia

**Keywords:** Overactive bladder, Incontinence, Nocturia, Urgency, Phytomedicine

## Abstract

**Background:**

Storage lower urinary tract symptoms (LUTS) including overactive bladder (OAB) and urinary incontinence (UI) affect millions of people worldwide, significantly impacting quality of life. Plant based medicines have been documented both empirically and in emerging scientific research to have varying benefits in reducing bladder symptoms. We assessed the efficacy of Urox®, a proprietary combination of phytomedicine extracts including, Cratevox™ (*Crataeva nurvala*) stem bark, *Equisetem arvense* stem and *Lindera aggregata* root, in reducing symptoms of OAB and UI.

**Methods:**

Efficacy of the herbal combination on a variety of bladder symptoms compared to an identical placebo, were documented in a randomised, double-blind, placebo controlled trial conducted at two primary care centres. Data were collected at baseline, 2, 4 and 8 weeks, with the primary outcome being self-reported urinary frequency. Statistical analysis included mixed effects ordered logistic regression with post hoc Holm’s test to account for repeated measures, and included an intention-to-treat analysis.

**Results:**

One hundred and fifty participants (59% female, aged; mean ± SD; 63.5 ± 13.1 years) took part in the study. At week 8, urinary day frequency was significantly lower (OR 0.01; 95%CI 0.01 to 0.02; *p* < 0.001) in response to treatment (mean ± SD; 7.69 ± 2.15/day) compared to placebo (10.95 ± 2.47/day). Similarly, episodes of nocturia were significantly fewer (OR 0.03; 95%CI 0.02 to 0.05) after 8 weeks of treatment (2.16 ± 1.49/night) versus placebo (3.14 ± 1.36/night). Symptoms of urgency (OR 0.02; 95%CI 0.01 to 0.03), and total incontinence (OR 0.03; 95% CI 0.01 to 0.06) were also lower (all *p* < 0.01) in the treatment group. Significant improvements in quality of life were reported after treatment in comparison to placebo. No significant side effects were observed resulting in withdrawal from treatment.

**Conclusions:**

The outcome of this study demonstrated both statistical significance and clinical relevance in reducing symptoms of OAB, urinary frequency and/or urgency and incontinence. The demonstrated viability of the herbal combination to serve as an effective treatment, with minimal side-effects, warrants further longer term research and consideration by clinicians.

**Trial registration:**

NCT02396160 (registered on 17 March 2015 - before any statistical analyses commenced).

## Background

Lower urinary tract symptoms (LUTS) are subjective and divided into subgroups with the three major groups as storage, voiding and post micturition symptoms. Storage LUTS include increased day frequency, nocturia, urgency, and urinary incontinence. Urinary incontinence can be subdivided further into stress, urgency, a mixture of both stress and urgency, and the less common, enuresis, continuous and other types such as situational incontinence [1]. Voiding LUTS include slow stream, splitting/spraying, intermittency, straining and terminal dribble. Post micturition symptoms include feeling of incomplete emptying and post micturition dribble. Storage LUTS include Overactive bladder (OAB) and urinary incontinence (UI) and are embarrassing and distressing conditions suffered by millions. These conditions can negatively impact the physical and emotional status and quality of life in those affected [2–4]. OAB is characterized by symptoms of urinary urgency, with or without leakage (urgency incontinence), day frequency, and nocturia. Urinary incontinence may also be described as occurring due to stress (stress incontinence), where pressure is placed on the pelvic floor muscles such as in cases of sneezing or heavy lifting, where the urinary loss occurs without warning or the sense of urgency beforehand (urgency incontinence) or a mixture of both [2].

Current estimates suggest 4.8 million Australians suffer with urinary incontinence, while 15% of the population experience OAB [5]. It is estimated that 33 million Americans suffer from OAB with 12.2 million of these adults also having concomitant urgency incontinence [2, 6]. A 2002 study of 5 other western countries (i.e., Canada, Germany, Italy, Sweden and the United Kingdom) demonstrated that on average, OAB prevalence was around 11.8% and UI 9.4% [7]. These conditions pose an enormous health cost burden. In 2000, these costs totalled US$19.5 billion for urinary incontinence alone in the United States [8].

Contrary to popular understanding, men and women have a similar prevalence of overactive bladder, with urgency incontinence occurring more often in males [2, 6, 9]. Less than 50% of men with lower urinary tract symptoms (LUTS) have urodynamically proven bladder outlet obstruction that may be attributed to BPH or other obstructive causes [10, 11]. Men with BPH may be prescribed phosphodiesterase type 5 (PDE5) inhibitors such as tadalafil, although the more commonly prescribed medications for male LUTS attributed to BPH are alpha-adrenergic agents to relax the urethral and prostate smooth muscle and/or 5-alpha reductase inhibitors to reduce androgen production and prostate growth. Side effects from these two classes of medications include hypotension, erectile dysfunction and/or reduced libido. For the 50% of men with LUTS due to storage problems, predominantly OAB, their response to these medications is limited [1].

Combined, stress and urgency UI affects approximately 25% of reproductive age women, 50% of post-menopausal women, and 50%–75% of women in nursing homes [3, 12]. Despite this, UI remains under-diagnosed and under-reported [3] with many women perceiving UI as a ‘normal’ part of being female [13]. Factors contributing to urinary incontinence include, child birth (40% of women suffer some form of leaking urine after bearing children) [14], constipation and other bowel symptoms, a high body mass index, as well as, a history of hysterectomy, prostatectomy or prolapse repair [15].

Current popular treatment options for UI and OAB include pelvic muscle rehabilitation, behavioural techniques such as bladder retraining, and drug therapies [9]. Common drugs used in the treatment of UI and OAB are oxybutynin, tolterodine and fesoterodine, anticholinergic muscarinic receptor antagonists, intended to reduce symptoms of OAB and UI with variable outcomes [16, 17]. However, some anticholinergic drugs can effect tissues other than the bladder resulting in poorly tolerated side effects including dry mouth, dry eyes, constipation and memory loss [18], and increased discontinuation of treatment within a two to twelve month timeframe [19]. Other reported side effects include blurred vision, nausea and drowsiness [20]. Recent research has documented anticholinergic and antimuscarinic drugs, including high dose oxybutynin, may produce irreversible cognitive impairment and an increased risk for dementia, particularly with chronic use [21]. This has led to recent recommendations to minimise anticholinergic use, in general, over time [22, 23]. Despite recent approval of additional pharmaceutical medications which are more specific to bladder tissue for the treatment of OAB and UI, such as darifenacin, solifenacin and trospium, these have not been reported to date to result in cognitive impairment, however, unwanted anticholinergic effects in tissues other than the bladder continue to be reported. Even administration of slow release or transdermal delivery systems have not eliminated unwanted systemic anticholinergic side effects [24].

Phytomedicine therapies have been traditionally used in the treatment of symptoms of OAB and UI [25–28]. Both previous and emerging clinical research into the use of phytomedicines demonstrates an increasing benefit from these approaches, often without the side effects that are associated with prescription medications. However, few have been subjected to controlled clinical trials to evaluate their safety and efficacy. The specific phytomedicines used in the herbal combination, *Crataeva, Equisetum* and *Lindera*, have well established traditional uses [25–27] and as such considered safe for human consumption [29].

In Ayurvedic Medicine, *Crataeva nurvala* is the herb of choice for urinary disorders [30]. A review of research shows *Crataeva* produced hypertonic curves in dog cystometric studies and increased bladder tone and bladder capacity in humans in cases of hypotonic bladder due to prostatic hypertrophy [28]. *Crataeva* demonstrated beneficial effects on neurogenic bladder and significantly decreased residual urine volume, normalising the tone of the urinary bladder. Crataeva has also been shown to be effective in the treatment of urinary calculi and infection [28, 31–35].

Western herbal medicine traditionally recommends *Equisetum arvense* as a genito-urinary astringent for urinary incontinence and enuresis in children [27]. The silica content of *Equisetum* likely contributes to the astringent effects. *Equisetum* has also been shown to have anti-inflammatory, anti-bacterial and anti-lithogenic effects [27, 36–38]. A pilot trial with *Equisetum* and *Crataeva* showed this combination reduced urinary frequency, urgency incontinence and stress incontinence episodes, which was attributed to improved tone of the urinary bladder and pelvic floor [39]. A randomised controlled trial with *Crataeva* and *Equisetum* alone, showed statistically significant reductions in day frequency and urinary incontinence and improved quality of life within two months of treatment, however, drop-out was high (23%) [29] in addition, Human cytochrome P450 (CYP1A2 and CYP3A4) in vitro testing on immortalised human hepatocytes (Fa2N-4 cells) showed that the combination of *Crataeva* and *Equisetum* caused no interference with these liver enzymes involved in drug metabolism, indicating that the combination of the two herbs was safe when consumed with other medications [40]. *Lindera aggregata,* another herb, is documented in texts of traditional Chinese medicine for frequent urination and urinary incontinence due to cold from a deficient bladder [25]. *Lindera* promotes the movement of chi or energy and blood and disperse cold, especially in the lower abdomen [25].

Urox (herbal combination used in the current study) contains *Crataeva, Equisetum* and *Lindera*. Urox is listed as safe for human consumption with the Therapeutic Goods Administration (TGA) in Australia since 2011 and is being sold in the USA and Australia since 2012. Company documentation (personal communication with Seipel Group) indicate less than 0.07% of adverse event (predominantly worsening bladder symptoms, digestive complaints and allergy to the formula) between 2012 and 2017 with no report of any interference with drug medications. It is speculated that the efficacy of Urox to be due to an antispasmodic effect as a result of an improvement in the tone of the bladder’s muscles [28, 29]. Urox was intended as an improved formulation with a faster action, therefore results from the current study are required within the two-month timeframe to assess its efficacy in comparison to the previous formulations [29, 39].

As pharmaceutical treatment options for UI and OAB have limited effectiveness and are associated with a range of adverse side effects, and given the paucity of competent and reliable studies of phytomedicine treatments to substantiate efficacy and/or safety, we assessed the effectiveness of Urox, the herbal combination of *Crataeva, Equisetum* and *Lindera* towards resolving UI and/or symptoms of OAB, such as urinary frequency and urgency within a two-month time frame.

## Methods

This study was conducted over an 8-week period in a phase-2, parallel double-blinded, randomised controlled design. Adults over the age of 18 years with symptoms of UI and/or OAB were recruited via a variety of advertising media including newspapers advertisements and notices posted at community centres. Self-identified participants were initially screened for suitability via telephone by research clinicians, based on definitions outlined by the Standardization Committee of the International Continence Society.

### Ethics, consent and permissions

The study was approved by the Ethics Committee of Endeavour College of Natural Health (Queensland, Australia; approval number HREC #12/030). All participants provided written informed consent.

Inclusion criteria, based on an adult only population, included those who experienced in the most recent six months, symptoms such as: urinary day frequency (≥10/day), nocturia (≥2/night), urgency (≥2/day), and incontinence (≥1/day). To be eligible, participants needed to have a minimum of 2 of these symptoms. Urodynamics were not performed, patients were recruited solely on the basis of their symptoms, as the former is invasive and provides only a brief snapshot of bladder function under artificial conditions [41]. Participants with comorbidities such as controlled hypertension, osteoarthritis, controlled diabetes, anxiety, chronic obstructive pulmonary disease, etc., were included in the study. These diseases/disorders were not expected to confound the results.

Exclusion criteria included: recent (≤1 year) relevant surgeries such as hysterectomy, prolapse repair, prostate surgery, childbirth/currently pregnancy; current use of any natural therapies for bladder symptoms or prescribed medication for UI or OAB; unregulated doses of diuretics; undergoing treatment for mental health issues or psychiatric disturbances; other concomitant health conditions, including uncontrolled diabetes mellitus, heart disease, pancreatic, hepatic or renal disease, neurologic disease, recurrent urinary tract infections, benign prostatic hypertrophy, continual leakage, menstrual cycle-related incontinence, and chronic inflammatory conditions.

### Randomisation

Participants meeting the above criteria, provided written informed consent and were randomised via the block of four method (using Microsoft Excel® command “Rand”) by a third party, into either treatment or placebo as indicated by either blue or yellow stickers on identical product bottles and allocated patient files. Both participants and researchers remained blinded to treatment allocation until after completion of statistical analyses, to ensure no risk of bias for the entire duration of the study and into completion. Treatment allocation was provided by a 3rd party external to the researchers and clinicians and was not disclosed until after statistical analysis, also minimising the chance of bias.

### Interventions

Each capsule contained 420 mg of a proprietary blend of Urox® (Seipel Group, Brisbane, Australia) containing Cratevox™ (*Crataeva nurvala* L.; Capparidaceae; Varuna) stem bark extract standardised for 1.5% lupeol; non-standardised *Equisetum arvense* L. (Equisetaceae; horsetail) stem extract; and, non-standardised *Lindera aggregata* Sims. (Lauraceae; Japanese evergreen spicebush) root extract. The placebo contained a colour-matched vegetarian capsule containing colour-matched cellulose. Identity of each plant was confirmed prior to manufacture of the finished capsule via high performance thin layer chromatography (HPLC) for *Crataeva* and *Equisetum* (Southern Cross University, Lismore, New South Wales, Australia) and *Lindera* (Alkemist Labs, Costa Mesa, California, USA). *Crataeva* and *Equisetum* were wild-crafted and grown without the use of pesticides. *Lindera* was cultivated and was tested for pesticide residue by gas chromatography mass spectroscopy (GC-MS).

Capsules were manufactured in a Therapeutic Goods Administration licensed facility according to the PIC/S Guide to Good Manufacturing Practice for Medicinal Products, PE 009–9-15 January 2009. Capsules underwent microbiological and heavy metal testing to ensure they complied with product specifications. The dosage was two capsules per day taken once daily with food. The dosage regimen was determined based on earlier research with *Crataeva* and *Equisetum* alone [29], pharmacopeia and traditional herbal textbook dosage recommendations, and earlier, unpublished research with the *Crataeva, Equisetum* and *Lindera* blend [25–28].

Initial consultations were held at two Brisbane outpatient centres. Follow-up interviews were conducted via telephone. Participants were telephoned approximately one week before the scheduled interview and were intermittently called during the week thereafter if they were unable to be reached to schedule an alternative interview time. The clinicians conducting interviews were herbalists with additional qualifications in naturopathic medicine, acupuncture and/or traditional Chinese medicine. In Australia, herbalists are regulated either by the National Herbal Association of Australia (NHAA) or, if the herbalist has multiple qualifications which is often the case, they are regulated by other professional association bodies (e.g., the Australian Acupuncture and Chinese Medicine Association, the Australian Naturopathic Practitioners Association, etc.).

The week prior to initial consultation participants were requested to complete a micturition diary and relevant health related quality of life surveys. The attending clinician completed a clinical data sheet, containing a range of questions including demographics, exercise, health history and personal habits. Any incomplete quality of life surveys at outset were completed during the initial interview. Micturition diaries were collected by post at 2, 4 and 8 week intervals, along with the completion of quality of life surveys and follow up clinical data sheet via telephone. Participants were asked to keep the micturition diary for 3 days prior to each consultation and were provided reply paid envelopes to return the surveys and any unused capsules to assess compliance.

The primary outcome measure was urinary frequency defined as the number of voluntary diurnal and/ or nocturnal micturitions, self-reported via a validated urinary diary method per Wyman [42]. These diaries are designed to assist with the collation of urinary symptoms for 3 consecutive days, where participants indicate the number of times each symptom occurs. Relevant symptoms include urinary frequency, urgency, urgency incontinence or stress incontinence. Long-term diaries decrease patient compliance while a 3-day diary is reported as reliable to that of a 7-day record [43]. Secondary outcome measures included number of urinary urgency episodes/day, and number of incontinence episodes/day, using the same diary technique as for the primary measure.

Health-related quality of life (HR-QOL) was measured using condition specific, previously validated standardised instruments outlined elsewhere [44–47]. These surveys included the short versions of the Overactive Bladder Questionnaire (OAB-SF; maximum score for each question = 6), which was administered if symptoms of urgency and/or frequency existed, while the Urinary Distress Inventory (UDI; maximum score for each question = 4) and Incontinence Impact Questionnaire (IIQ; maximum score for each question = 4) were utilised in cases of urinary incontinence.

Information regarding number and type of incontinence pad/diaper usage per week was collected from all participants at each time point. At the completion of the trial participants were asked if they found any benefit from the treatment and whether they would be willing to continue treatment with this medication.

Analysed outcome data was collected via patients’ self-evaluation and returned by patients via mail in order to minimise clinicians and researchers chance to elicit any hypothetical bias and influence outcomes. Once received, diary responses were filed until data entry and subsequent analyses. Participants were not patients of the interviewing clinicians and their postal outcome results were not considered or evaluated for the duration of the trial. These follow up interviews consisted of quality of life questions as well as questions to ascertain any changes in participants’ lifestyle, which might otherwise confound results such as changes in exercise, water intake, diet or extraneous stress, or newly presenting medical conditions and medication changes.

### Sample size calculation and statistical analysis

For the primary outcome (day frequency), based on earlier research using a *Crataeva* and *Equisetum* combination [29], it was calculated that 45 participants in each of the 2 groups were required to detect a difference of 1.6 (±2.7) urinary frequency episodes per day between treatment and placebo groups, with a two-tailed alpha of 0.05 and a power of 80%. For total incontinence, 53 participants were required to detect a difference of 1.2 (±2.2) incidents/day, while for urgency 54 participants were required in each group to detect a difference of 2 (±3.7) incidents/day. To account for potential drop-outs and variations in presenting symptoms, a total of 150 participants (75/group) were recruited. Participants were enrolled if they presented with at least two of the four assessable symptoms: day frequency, urgency, nocturia, and/or incontinence.

One person was responsible for data transfer from paper copies to the electronic database. Although double data entry was not performed, 10% of the data was randomly checked until no mistakes were found. Less than 1% changes were made to the data set following this procedure. All data except for day frequency was non-normally distributed. Residuals (difference between predicted and actual observed value) of linear regression model for all variables did not meet the assumptions of linear regression. Hence, all comparisons for the two treatments (as change from baseline) were made using mixed effects ordered logistic regression adjusted for repeated measures (Stata, v. 13.1, StataCorp, TX, USA) as this model does not require the assumptions of linear regression to be true. Holm estimation test was used to adjust *p*-values for repeated measures. Analyses included only participants who were symptomatic at baseline for each parameter investigated. Data (day frequency, nocturia, urgency and incontinence) were evaluated by per-protocol and intention-to-treat analysis, with the last result brought forward for participants who dropped out or were lost to follow-up. Backward stepwise regression (*p* < 0.22 for covariate inclusion) indicated small effect of age, gender, water intake and diuretic use on the outcome model. However, result for adjusted and non-adjusted data were similar, hence unadjusted analyses is presented.

Responses to questionnaires were grouped for ease of reporting. The OAB-SF was analysed if the participant experienced urgency at baseline. The change from baseline is reported for the total of all OAB-SF questions, as well as sub-grouped according to classifications of symptom bother (questions 1–6; maximum score 36); difficulty coping (questions 7, 10, 11, 14, 19; maximum score 30); concern/worry (questions 8, 12, 16; maximum score 18); interference in sleep (questions 9, 13, 17; maximum score 18) and affects social interaction (questions 15, 18; maximum score 12). The IIQ questions 1–7 (maximum score 28) are reported as a total as are the responses to UDI questions 8–13 (maximum score 24).

## Results

**Fig. 1:**
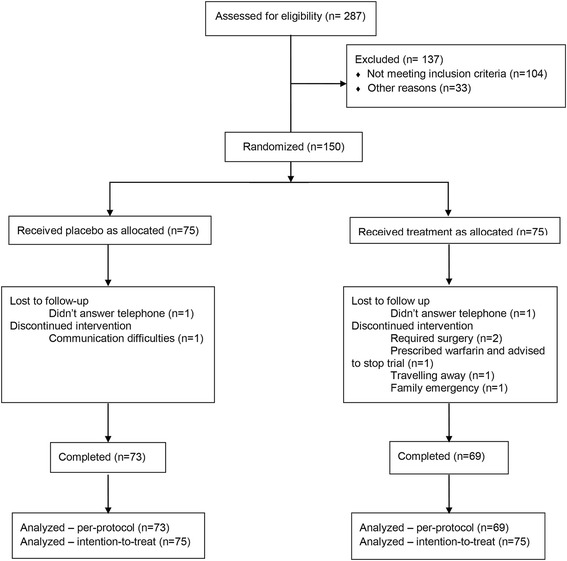
Participant flow through the study

Participants were recruited and data collected from April 2013 until November 2014. One hundred and fifty participants consented to take part in the study (Fig. 1), and 142 (73 (97%) placebo, 69 (92%) treatment) completed the study. Drop-outs included two participants from the placebo group and six from the treatment group. None of these withdrawals were due to the study interventions but were for personal reasons. None of the adverse events reported were severe enough to cause withdrawal. Baseline demographic and symptom characteristics of placebo and treatment groups were similar between the two groups (Table 1).

**Table 1:** Demographic and symptom characteristics of study population at the start of the trial

Variable	Placebo	Treatment
Participants	75	75
Sex (Female %)	48 (64%)	40 (53%)
Age (years; mean ± SD)	62.48 ± 13.70	64.55 ± 12.42
Weight (kg; mean ± SD)	79.23 ± 22.47	78.53 ± 18.94
Day frequency ≥ 10 (n,%)	57 (76%)	50 (67%)
Nocturia ≥2 (n,%)	60 (80%)	70 (93%)
Urgency ≥2 (n,%)	62 (83%)	63 (84%)
Urgency incontinence≥1 (n,%)	33 (44%)	32 (43%)
Stress incontinence ≥1 (n,%)	9 (12%)	10 (13%)
Any incontinence ≥1 (n,%)	42 (56%)	35 (47%)
Only two symptoms (n,%)	25 (30%)	26 (35%)
Only three symptoms (n,%)	29 (39%)	30 (30%)
All four symptoms (n,%)	21 (28%)	19 (25%)

Symptoms include urinary day frequency (≥10/day), nocturia (≥2/night), urgency (≥2/day), and incontinence (≥1/day) for ≥6 months prior to enrolling in the study; any incontinence includes urgency, stress and other incontinence

**Fig. 2:**
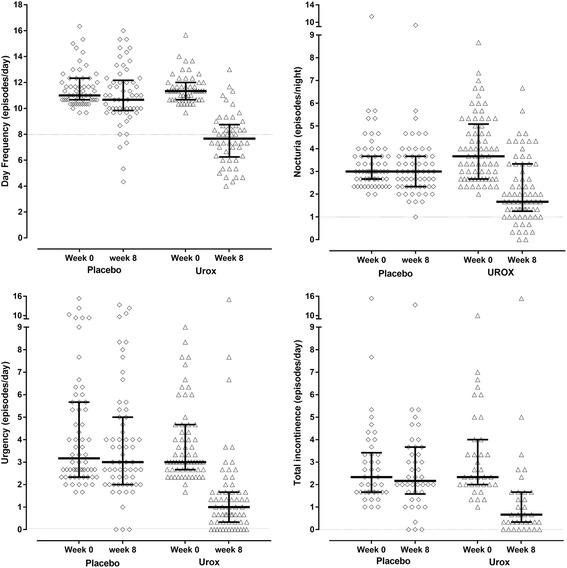
Frequency of urinary symptoms averaged over three consecutive days (median, 25 and 75 percentile)

Significant differences were observed at 8-weeks over all variables, with symptoms reducing to within normal range occurring in greater frequency in the treatment group (Table 2). Resolution of symptoms was indicated at ≤8/day for day frequency, ≤ 1/night for nocturia and 0 for both urgency and total incontinence (Fig. 2), as outlined by the National Association for Continence [2].

**Table 2:** Over active bladder and urinary incontinence symptoms frequency as recorded from micturition diaries

Variable	Placebo (mean ± SD)	UROX (mean ± SD)	OR (95% CI) Placebo vs treatment
Day frequency (n/day)			
Week 0	11.57 ± 1.79	11.53 ± 1.54	0.95 (0.33 to 2.73)
Week 2	10.80 ± 2.44	8.94 ± 2.28	0.07 (0.04 to 0.13)^*^
Week 4	10.60 ± 2.42	8.42 ± 2.46	0.04 (0.02 to 0.08)^*^
Week 8	10.95 ± 2.47	7.69 ± 2.15	0.01 (0.01 to 0.02)^*^
Nocturia (n/day)			
Week 0	3.39 ± 1.52	4.02 ± 1.62	3.59 (1.39 to 9.21)^*^
Week 2	2.94 ± 1.37	3.18 ± 1.72	0.40 (0.24 to 0.69)^*^
Week 4	2.92 ± 1.30	2.70 ± 1.52	0.14 (0.08 to 0.24)^*^
Week 8	3.14 ± 1.36	2.16 ± 1.49	0.03 (0.02 to 0.05)^*^
Urgency (n/day)			
Week 0	4.34 ± 2.89	3.80 ± 1.82	0.67 (0.23 to 1.94)
Week 2	3.65 ± 2.62	2.32 ± 2.09	0.16 (0.09 to 0.27)^*^
Week 4	3.52 ± 2.68	1.88 ± 2.25	0.08 (0.04 to 0.13)^*^
Week 8	3.93 ± 2.87	1.49 ± 2.31	0.02 (0.01 to 0.03)^*^
Urgency Incontinence (n/day)			
Week 0	2.71 ± 2.68	2.79 ± 1.50	1.70 (0.53 to 5.40)
Week 2	2.32 ± 1.54	1.85 ± 1.78	0.19 (0.09 to 0.40^)*^
Week 4	1.82 ± 1.33	1.53 ± 2.41	0.19 (0.09 to 0.40)^*^
Week 8	2.44 ± 2.38	1.24 ± 2.49	0.04 (0.02 to 0.09)^*^
Stress Incontinence (n/day)			
Week 0	2.19 ± 1.50	2.13 ± 1.14	0.97 (0.11 to 8.65)
Week 2	1.70 ± 1.49	1.27 ± 1.29	0.30 (0.07 to 1.29)
Week 4	1.85 ± 1.29	0.77 ± 0.94	0.06 (0.01 to 0.25)^*^
Week 8	2.04 ± 1.51	0.73 ± 0.87	0.03 (0.01 to 0.15)^*^
Total Incontinence (n/day)			
Week 0	2.95 ± 2.65	3.31 ± 2.12	1.97 (0.65 to 5.98)
Week 2	2.56 ± 1.62	2.20 ± 2.09	0.23 (0.11 to 0.45)^*^
Week 4	2.13 ± 1.42	1.74 ± 2.68	0.14 (0.07 to 0.27)^*^
Week 8	2.70 ± 2.25	1.38 ± 2.73	0.03 (0.01 to 0.06)^*^

OR (95% CI): odds ratio and 95% confidence interval for difference between the two treatments (includes participants who did not complete the study); Comparison made as change from baseline between the two conditions. Holm estimation test was used to adjust *p*-values for repeated measures

^*^Significantly different between the two treatments at the specific time; Number of participants assessed for each symptom were according to if their baseline measures matched the inclusion criteria (see Table 2 for the exact number of participants)

**Fig. 3:**
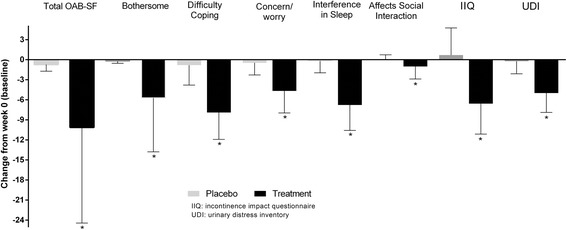
HR-QOL scores at week-8 on placebo and the Urox treatment. *Significantly different from placebo when compared as change from baseline

Baseline results for all HR-QOL variables were similar between the two intervention groups (all *p* > 0.18). Significant improvements (all *p* < 0.001) were noted in participants’ perceptions after 8-weeks of the treatment compared to placebo (Fig. 3).

Differences in OAB-SF parameters (60 in placebo and 58 in treatment group) between the herbal combination and placebo (analysed as change from baseline) for those with symptoms of urgency at baseline included: bothersome (mean diff − 11.53; 95% CI -13.15 to − 9.90); difficulty in coping (− 7.57; − 8.71 to − 6.43), concern/worry (− 4.41; − 5.30 to − 3.52), difficulty sleeping (− 6.51; − 7.58 to − 5.44) and social interaction (− 1.14; − 1.59 to − 0.68) were all significantly lower (*p* < 0.01 based on logistic regression analyses) at the end of treatment compared to placebo. Total OAB-SF was significantly lower (− 30.83; − 34.75 to − 26.93; *p* < 0.001) after Urox than with placebo. Total scores for IIQ for those with incontinence at baseline were also significantly lower (− 7.56; − 9.30 to − 5.82) after the herbal combination than placebo. UDI responses for all participants were also significantly lower (− 5.45; − 6.65 to − 4.25) after 8-weeks of Urox, compared to placebo.

To assess whether the differences observed between the placebo and the Urox at week-8 were clinically relevant, we calculated the Cohen’s *d* effect size for each variable using week-8 values. Results showed a large (Cohen’s *d* ≥ 0.6) effect of the herbal combination on all urinary variables expect for urgency incontinence where the effect was moderate (Cohen’s *d* 0.55). Similarly, for HR-QOL data, we observed Cohen’s *d* value of above 0.6.

Thirteen cases of transient adverse events were reported, eight from the placebo arm and five from the treatment group (Table 3).

**Table 3:** Adverse Events Reported

Symptom	Placebo	UROX
Episodes of diarrhoea	2	2
Urinary tract infection	1	2
Headache and worsened asthma	1	
Worsened memory	1	
Flatulence		1
Facial flushing	1	
Halitosis	1	
Worsened arthritic pain	1	

Comparison of number of pads used and the type of pad used showed significant differences (both *p* < 0.001) at the end of the study when compared between the two interventions. Approximately 40% of the participants in both groups (29/72 placebo; 26/69 Urox) reported using pad/diapers at the start of the study. Considering the use of 7 or less pads per week (≤1/day) as an indication of pad use as a precautionary measure, 20 out of 72 and 16 out of 69 participants reported using more than one pad/diaper a day (i.e. > 7/week) at the start of the study (week-0), in placebo and treatment groups respectively. Nineteen participants in the placebo group continued to use more than one pad/diaper a day at the end of the study, compared to only four in the Urox group. The number of individuals using moderate/heavy pads changed from 20 at baseline to 19 at the end of the study period in the placebo group, whereas, only six participants reported using moderate/heavy pads at week-8 in the Urox group, a reduction from 18 at baseline. Similarly, significantly higher proportion (*p* < 0.001) of participants in the Urox group (84%; 58/69) reported they had benefited from the treatment compared to the placebo group (18%; 13/72). Furthermore, 77% (53/69) in the treatment group compared to 29% (21/72) in the placebo group indicated their willingness to continue with “this medication” (*p* < 0.001).

## Discussion

This study demonstrated significant efficacy and tolerability of the phytomedicine formula, Urox, in the treatment of UI and symptoms of OAB. A strength of this study is the lack of side effects typically associated with anticholinergic/antimuscarinic medications over the same 8-week period and the significant improvement in quality of life.

Our results show that 6 out of 57 (11%) symptomatic participants from the placebo group reported normalization of their bladder symptoms for day frequency after week-8 of the trial, compared with 30 out of 50 (60%) symptomatic participants in the treatment group. Similarly, for nocturia, 17 of 70 symptomatic participants (24%) in the treatment group became symptom free at the end of the trial compared to only 1 of 60 participants (less than 2%) in the placebo group. Five of these 17 participants in the treatment group reported reversal of the condition at week-2 and remained asymptomatic to the end of the trial. Twenty percent (13/63) of participants (with baseline urgency of 2 or more episodes a day) in the treatment group reported urgency of zero at the end of the trial period, compared to only three participants (out of 62) in the placebo group. Twenty-three percent (8/35) of the participants in the Urox group reported to be incontinence free at the end of 8 weeks compared to 7 % in the placebo group (3/42).

Urox produced more extensive benefits for symptoms of OAB and UI than has been shown with earlier herbal research, and within a shorter timeframe. A 12-week randomised trial with women using a pumpkin seed extract (*Cucurbita pepo.*) and a proprietary soy germ extract (*Glycine max*) combination showed no significance in reducing incontinence [48]. Whilst frequency, urgency and nocturia improved, the magnitude of effect was consistently less and time frame for results, longer than with Urox. An 8-week randomized trial with men using the herb, *Angelica archangelica* was assessed for its effects on nocturnal bladder symptoms and showed no significant effect in overall nocturia [49]. Research with whole cranberry (*Vaccinium macrocarpon)* fruit powder showed reductions in urinary urgency, frequency and nocturia, but again took longer (three to six months) than demonstrated in this study of Urox [50].

A systematic literature review of pharmaceutical drugs for urgency and urinary incontinence between 1966 and 2011, concluded that overall the drugs selected for review produce small benefits, with up to 13% of participants achieving continence, while approximately 6% of participants discontinued treatment due to severity of adverse effects. Also, evidence of improved quality of life was limited [24]. In contrast, the current study using Urox, compared to placebo, was well tolerated and significantly improved quality of life for study participants.

Halving nocturia within two months of treatment reduces the additional debility of interrupted sleep in these patients, and is considered highly clinically relevant. Reductions in day urinary frequency, urgency and incontinence are also very clinically relevant particularly to patients whose lives can be continuously interrupted on a daily basis by needing to use toilet facilities within a close proximity of their whereabouts. Considering the demonstrated benefits, lack of serious adverse events, along with a high rate of patient compliance and participant satisfaction, the utility of Urox by clinicians seems worthy of consideration.

As this is a phase-2 clinical trial of 8 weeks duration without an active comparator, and given the results reported, a longer phase-3 trial is warranted.

## Conclusion

The outcome of this randomised controlled trial demonstrates both statistical significance and clinical relevance in reducing urinary frequency, OAB and UI symptoms, over 8 weeks, without side effects commonly seen with anticholinergic and antimuscarinic medications.

## Abbreviations

HR-QOL: Health-Related Quality of Life
IIQ: Incontinence Impact Questionnaire
LUTS: Lower Urinary Tract Symptoms
OAB: Overactive Bladder
OAB-SF: Overactive Bladder Short Form
UDI: Urinary Distress Inventory
UI: Urinary Incontinence

## References

[1] Abrams P, Cardozo L, Fall M, Griffiths D, Rosier P, Ulmsten U (2003). The standardisation of terminology in lower urinary tract function: report from the standardisation sub-committee of the international continence society. Urology.

[2] Conditions. http://www.nafc.org/conditions/. Accessed Nov 2016.

[3] Culligan P, Sand P (1998). Involuntary urine loss in women: help for a hidden problem. Patient Care.

[4] Bartoli S, Aguzzi G, Tarricone R (2010). Impact on quality of life of urinary incontinence and overactive bladder: a systematic literature review. Urology.

[5] Who's at risk? http://www.continence.org.au/pages/the-facts.html. Accessed Nov 2016.

[6] Stewart W, Van Rooyen J, Cundiff G, Abrams P, Herzog A, Corey R, Hunt T, Wein A (2003). Prevalence and burden of overactive bladder in the United States. World J Urol.

[7] Irwin D, Milsom I, Hunskaar S, Reilly K, Kopp Z, Herschom S, Coyne K, Kelleher C, Hampel C, Artibani W (2006). Population-based survey of urinary incontinence, overactive bladder, and other lower urinary tract symptoms in five countries: results of the EPIC study. Eur Urol.

[8] CDC (2014). US toll from incontinence. JAMA.

[9] Eslami M. Evaluation and Management of Incontinence in females. http://www.med.ucla.edu/modules/xfsection/article.php?articleid=93. Accessed Nov 2016.

[10] Hann-Chorng K (2010). Male lower urinary tract symptoms - an old problem from a new perspective. Incont Pelvic Floor Dysfunct.

[11] Chun-Hou L, Hann-Chorng K (2010). Measurement of international prostate symptom score subscores in male lower urinary tract symptoms. Incont Pelvic Floor Dysfunct.

[12] Chutka D, Fleming K, Evans M, Evans J, Andrews K (1996). Urinary incontinence in the elderly population. Mayo Clin Proc.

[13] Peake S, Manderson L, Potts H (1999). Part and parcel of being a woman: female urinary incontinence and constructions of control. Med Anthropol Q.

[14] Rane A (1999). Incontinence in women. Don’t suffer it. Aust Fam Physician.

[15] Chiarelli P, Brown W (1999). Leaking urine in Australian women: prevalence and associated conditions. Womens Health.

[16] Herschorn S, Jones J, Oelke M, MacDiarmid S, Wang J, Guan Z (2010). Efficacy and tolerability of fesoterodine in men with overactive bladder: a pooled analysis of 2 phase III studies. Urology.

[17] Madhuvrata P, Cody J, Ellis G, Herbison G, Hay-Smith E. Which anticholinergic drug for overactive bladder symptoms in adults. Cochrane Database Syst Rev. 2012;1:CD005429. 10.1002/14651858.CD005429.pub2 PMC12989262 22258963

[18] Hesch K (2007). Agents for treatment of overactive bladder: a therapeutic class review. Proc (Bayl Univ Med Cent).

[19] Wagg A, Compion G, Fahey A, Siddiqui E (2012). Persistence with prescribed antimuscarinic therapy for overactive bladder: a UK experience. BJU Int.

[20] Getsios D, Caro J, Ishak K, El-Hadi W, Payne K, O'Connel M, Albrecht D, Feng W, Dubois D (2004). Oxybutynin extended release and Tolterodine immediate release: a health economic comparison. Clin Drug Investig.

[21] Gray S, Anderson M, Dublin S, Hanlon J, Hubbard R, Walker R, Yu O, Crane P, Larson E (2015). Cumulative use of strong anticholinergics and incident dementia. JAMA Intern Med.

[22] Fox C, Richardson K, Maidment I (2011). Anticholinergic medication use and cognitive impairment in the older population: the Medical Research Council cognitive function and ageing study. J Am Geriatr Soc.

[23] Cai X, Campbell N, Khan B, Callahan C, Boustani M (2013). Long-term anticholinergic use and the aging brain. Alzheimers Dement.

[24] Shamliyan T, Wyman J, Ramakrishnan R, Sainfort F, Kane R (2012). Benefits and harms of pharmacologic treatment for urinary incontinence in women. Ann Intern Med.

[25] Bensky D, Gamble A. Chinese herbal: Materia Medica, revised edn. Seattle: Eastland Press Inc.; 1993.

[26] Bone K (1997). Clinical applications of Ayurvedic and Chinese herbs; monographs for the western herbal practitioner.

[27] BHP: British Herbal Pharmacopeia. 1983. Published by The Bristish Herbal Association, Lane House, Cowling, Nr. Keighley, West Yorks.

[28] Deshpande P, Sahu M, Kumar P (1982). Crataeva nurvala hook and Forst (Varuna) the Ayurvedic drug of choice in urinary disorders. Indian J Med Res.

[29] Schauss A, Spiller G, Chaves S, Gawlicka A. Reducing the symptoms of overactive bladder and urinary incontinence: results of a two-month, double-blind, placebo-controlled clinical trial. https://www.ics.org/Abstracts/Publish/44/000300.pdf. Accessed Oct 2016.

[30] Nadkarni KM (1976). Indian Materia Medica.

[31] Anand R, Patnaik GK, Roy K, Bhaduri AP. Antixoaluric and anticalciuric activity. Indian J Pharmacol. 1995;27(4):265–8.

[32] Varalakshmi P, Shamila Y, Latha E (1990). Effect of Crataeva Nurvala in experimental urolithiasis. J Ethnopharmacol.

[33] Malini M, Ramakrishnan B, Varalakshmi P (1995). Effet of Lupeol, a pentacyclic triterpene, on urinary enzymes in hyperoaluric rats. Jpn J Med Sci Biol.

[34] Geetha T, Varalakshmi P (2001). Anti-inflammatory activity of lupeol and lupeol linoleate in rats. J Ethnopharmacol.

[35] Geetha T, Varalakshmi P (1999). Anticomplement activity of triterpenes from Crataeva nurvala stem bark in adjuvant arthritis in rats. Gen Pharmacol.

[36] Council AB (2000). Horsetail herb. Expanded commission E monograph.

[37] Grases F, Melero G, Costa-Bauza A, Prieto R, March JG (1994). Urolithiasis and phytotherapy. Int Urol Nephrol.

[38] Nagao A, Seike M, Kobayashi H (1999). Inihibition of xanthine oxidase by flaonoids. Biosci Biotechnol Biochem.

[39] Steels E, Ryan J, Seipel T, Rao A (2002). Crataeva and equisetum reduce urinary incontinence symptoms. Aust Continence J.

[40] Schauss A, Steele E. Preliminary evidence of the safety and efficacy of urologic™, an herbal formulation for the treatment of urinary incontinence and overactive bladder. FASEB. 2006;20:A990.

[41] Norton P, Karram M, Wall L, Resenzeig B, Benson U, Fantl J (1994). Randomised double-blind trial of terodiline in the treatment of urge incontinence in women. Obstet Gynecol.

[42] Wyman J, Choi S, Harkins S, Wilson M, Fantl J (1988). The urinary diary in evaluation of urinary incontinence in women: a test-retest analysis. Obstet Gynecol.

[43] Nygaard I, Holcomb R (2000). Reproducibility of the seven-day in women with stress urinary incontinence. Int Urogynecol J Pelvic Floor Dysfunct.

[44] Coyne K, Zhou Z, Thompson C, Versi E (2003). The impact on health-related quality of life of stress, urge and mixed urinary incontinence. Br J Urol Int.

[45] Robinson J, Shea J (2002). Development and testing of a measure of health-related quality of life for men with urinary incontinence. J Am Geriatr Soc.

[46] Coyne K, Revicke D, Hunt T, Corey R, Stewart W, Bentkover J, Kurth H, Abrams P (2002). Psychometric validation of an overactive bladder symptom and health-related quality of life questionnaire: the OAB-q. Qual Life Res.

[47] Matza L, Thompson C, Krasnow J, Brewster-Jordan J, Zyczynski T, Coyne K (2005). Test-retest reliability of four questionnaires for patients with overactive bladder: the overactive bladder questionnaire (OAB-q), patient perception of bladder condition (PPBC), urgency questionnaire (UQ), and the primary OAB symptom questionnaire (POSQ). Neurourol Urodyn.

[48] Shim B, Jeong H, Lee S, Hwang S, Moon B, Storni C (2014). A randomized double-blind placebo-controlled clinical trial of a product containing pumpkin seed extract and soy germ extract to improve overactive bladder-related voiding dysfunction and quality of life. J Funct Foods.

[49] Sigurdsson S, Geirsson G, Gudmundsdottir H, Egilsdottir P, Gudbvjarnason S (2013). A parallel, randomized, double-blind, placebo-controlled study to investigate the effect of SagaPro on nocturia in men. Scand J Urol.

[50] Vidlar A, Vostalova J, Ulrichova J, Student V, Stejskal D, Reichenback R, Vrbkova J, Ruzicka F, Simanek V (2010). The effectiveness of dried cranberries (Vaccinium Macrocarpon) in men with lower urinary tract symptoms. Br J Nutr.

